# Histone deacetylase inhibitor-based chromatin precipitation for identification of targeted genomic loci

**DOI:** 10.14440/jbm.2018.216

**Published:** 2018-03-30

**Authors:** Thomas W. Hanigan, Jeanne M. Danes, Taha Y. Taha, Jonna Frasor, Pavel A. Petukhov

**Affiliations:** 1 Department of Medicinal Chemistry and Pharmacognosy, College of Pharmacy, University of Illinois, 833 South Wood Street, Chicago, IL 60612, USA; 2 Department of Physiology and Biophysics, College of Medicine, University of Illinois, 835 S. Wolcott Street, Chicago, IL 60612, USA

**Keywords:** histone deacetylase, chromatin immunoprecipitation, photoreactive probes

## Abstract

Histone deacetylase (HDAC) catalyzes the removal of acetyl marks from histones, effectively regulating gene expression. Genome wide chromatin immunoprecipitation (ChIP) studies have shown HDACs are present on numerous active and repressed genes. However, HDAC inhibitors (HDACi) only regulate a small subset of this population in a cell type dependent fashion. To determine genomic locations directly targeted by HDACi, we developed a chromatin precipitation method using a photoreactive HDAC inhibitor probe (photomate). We validate this method by analyzing several canonical HDACi regulated genes, CDKN1A and FOSL1, and compare it to traditional ChIP using HDAC1 antibodies. We show that HDACi target HDACs bound at the promoter regions but not gene bodies, differing from HDAC1 antibody-based ChIP in the case of CDKN1A. This approach is anticipated to be useful for genome wide studies to identify the subset of genes directly regulated by an HDACi in a given cell type.

## INTRODUCTION

Gene expression is regulated by post-translational modification of histone tails. In particular, histone acetylation is associated with condensation or relaxation of chromatin leading to a decrease or increase in transcriptional activity at particular genomic loci. Histone deacetylase (HDAC) is a family of 18 human enzymes, subdivided into four classes based on homology to yeast, that catalyze the removal of acetyl marks from histone tails. Aberrant recruitment of HDAC to particular genomic loci [1] and increased HDAC expression [2] are noted in several cancer types including those of the breast. Inhibitors of this family of enzymes have shown promise as anti-cancer therapeutics with several approved for the treatment of T-cell lymphoma or multiple myeloma [3].

Genome wide chromatin immunoprecipitation (ChIP) followed by high-throughput DNA sequencing (ChIP-seq) studies have shown HDACs are widely distributed, both on promoters and gene bodies of both active and repressed genes [4-6]. However, HDAC inhibitors (HDACi) only effect the expression of 2%–5% of the genome [7,8]. Furthermore, the set of genes regulated by an HDACi has been shown to be cell type dependent [9-13]. It has been hypothesized that the differences in gene expression contribute to the cell type dependent phenotypes induced by HDACi [12]. Given this cell type dependent variation in gene occupancy and associated phenotypic changes induced by HDACi, technology to quickly determine the subset of genes targeted by HDACi in a cell type of interest is needed. While genome wide microarray analysis in conjunction with treatment of HDAC inhibitors has been instrumental to determine the genes affected by HDACi [10], this method cannot exclude indirect regulation, through off target effects and secondary regulation in response to primary targeted genes, nor can it report on the specific isoform responsible for regulating the gene of interest. In addition, ChIP with HDACi treatment has been applied successfully to determine HDACi regulated genes [6]. However, HDACs often serve as scaffolding proteins, and gene occupancy is not dependent on catalytic activity in certain cases [14,15]. Distinguishing genes regulated by HDAC catalytic activity cannot be resolved with the current antibody-based ChIP methodology. In this paper, we validate a similar method using a photoreactive HDAC inhibitor probe (photomate) to identify genomic loci directly targeted by HDACi.

## MATERIALS AND METHODS

The synthesis of photomate is characterized elsewhere [16]. The synthesis of the photomate ester is outlined in the supporting information.

### Reagents

All chemical reagents were purchased from Sigma Aldrich or Thermo-Fisher unless stated otherwise. Primers were purchased from integrated DNA technologies (IDT) unless otherwise stated. Cell culture reagents were purchased from Sigma Aldrich.

### Cell culture

Human MCF-7 cells were obtained from Dr. Debra Tonetti (University of Illinois at Chicago) and were maintained in RPMI supplemented with 10% FBS, 1% non-essential amino acids, 2 mM L-glutamine, 1% antibiotics penicillin-streptomycin, and 6 ng/ml human recombinant insulin at 37°C in 5% CO_2_.

### Crosslinking and sonication

Five 10 cm plates were seeded with 0.8 × 10^6^ MCF-7 cells and grown to 90% confluence. Cells were washed with PBS (2 × 5 ml) followed by crosslinking with 2 mM disuccinimidyl glutarate for 20 min at RT. After 20 min, 37% formaldehyde was added (to final concentration of 1%) to the plate and the cells were crosslinked for an additional 15 min. Crosslinking was quenched with 2.625 M glycine, the solution was decanted, the cells were scraped from plate into Eppendorf tubes, spun down at 1000× *g* for 5 min at 4°C, the supernatant removed, and the cells resuspended in 1 ml hypotonic buffer (10 mM HEPES (pH 7.5), 10 mM KCl, 0.4% Igepal CA-630, Protease inhibitor cocktail (Roche) and phosphatase inhibitor cocktail (Fisher)) until cell walls were compromised (verified by microscopic inspection). The mixture was spun down at 1000× *g* for 5 min to provide the cytosol (supernatant) and nuclei (pellet). The nuclei were than homogenized in 500 μl sonication buffer (50 mM HEPES (pH 7.5), 150 mM NaCl, 1.5 mM MgCl_2_, 1% Igepal CA-630, 0.1% SDS, protease inhibitor cocktail and phosphatase inhibitor cocktail) and incubated for 1 h at 4°C on a rotating stand. Lysed nuclei were then sonicated with a Fisher Dismembrator Model 100 until DNA was 300–500 base pairs and centrifuged at 20000 g for 10 min at 4°C to provide sonicated chromatin.

### Antibody-based chromatin immunoprecipitation

Lysate (200 μl) from crosslinking and sonication were split into two samples, 10 μl from each sample was saved as input for comparison with immunoprecipitates and the remaining lysate was diluted to 250 μl with sonication buffer and immunoprecipated overnight at 4°C with anti-HDAC1 antibody (Abcam, ab7028) or rabbit IgG control antibody (Abcam, ab46540) according to manufacturer’s directions. Prior to the immunoprecipitation, the antibodies were pre-bound for 2 h at RT to 100 μl of protein A coupled magnetic beads (Dynabeads, Invitrogen) in PBS with 5% BSA.

After overnight incubation, supernatant was discarded and the beads were washed twice with 250 μl low salt buffer (2 mM ethylenediaminetetraacetic acid (EDTA), 20 mM HEPES, 0.1% SDS, 1% Igepal CA-630, 150 mM NaCl), and then washed twice with 250 μl LiCl buffer (1 mM EDTA, 10 mM HEPES, 0.1% SDS, 0.1% SDC, 250 mM LiCl). Finally, beads were washed twice with 250 μl TE buffer (10 mM Tris, 1 mM EDTA) and the DNA was eluted with 120 μl of 10% SDS. Samples were decrosslinked overnight at 65°C using an Eppendorf Mastercycler thermocycler. DNA was then purified by GeneJet PCR Purification Kit (Thermo Scientific) per the manufacturer’s instructions. The purified samples were then diluted 1:5, while the inputs were diluted 1:200. The HDAC1 associated DNA fragments were verified using real-time polymerase chain reaction (PCR) or qPCR. The qPCR was performed on an Applied Biosystems Step-One Plus Real-Time PCR system using the Fast SYBR Green qPCR Mastermix (Applied Biosystems):

ChIP qPCR primers are listed below:
GAPDH (forward) AAA AGC GGG GAG AAA GTA GG
GAPDH (reverse) CTA GCC TCC CGG GTT TCT CT
CDKN1A Intron1 (forward) GTG CCT GCC TAG ATC CTA GTC CT
CDKN1A Intron1 (reverse) GGA GAC ACA CTG GTA TGT TTG AA
CDKN1A Promoter (Millipore, CS200575)
CDKN1A Promoter (forward) CCC ACA GCA GAG GAG AAA GAA
CDKN1A Promoter (reverse) CTG GAA ATC TCT GCC CAG ACA
FOSL1 Promoter and Exon 1 primers were detailed in [4]
FOSL1 Promoter (forward) GTG CTA TTT TGT GGG AGC AG
FOSL1 Promoter (reverse) TGG TGT AAC TTC CTC GCC GC
FOSL1 Exon 1 (forward) GCATGTTCCGAGACTTCGGG
FOSL1 Exon 1 (Reverse) TGCTGGGCTGCCTGCGCTGC

PCR efficiencies for each primer were calculated using standard dilutions of crosslinked and sonicated cell lysate. Inputs and sample Ct values were corrected based on fold dilution and primer efficiency. Percent input for each DNA sequence was calculated by raising the primer efficiency to the change in Ct value of input and sample. The data represented is from three independent experiments, and each qPCR amplification evaluation was performed in triplicate.

### Photomate-based chromatin enrichment

Lysate (300 μl) from crosslinking and sonication was split into three fractions and 10 μl of each fraction was saved as input. Each fraction was then diluted to 250 μl with sonication buffer and 10 μM photomate, 10 μM photomate ester or DMSO was added and incubated for 30 min at RT. Samples were transferred to a 6-well plate and irradiated with 365 nM light (35 J/cm^2^). Each reaction was incubated with azide conjugated biotin according to 1.5× concentration of probe, TCEP (0.25 mM), TBTA (50 μM), and CuSO_4_ (0.50 mM) for 90 min at RT. Samples were then left at **–**20°C overnight. Precipitated protein was spun down at 6000× *g* for 4 min at 4°C, and resuspended with brief sonication in 1 ml cold methanol. This was repeated twice and the pellets were resuspended in 1 ml 0.2% SDS in PBS by brief sonication and 10 min of heating at 60°C. Next, 30 μl of Dynabeads M-280 (ThermoFisher) were washed twice with 0.2% SDS in PBS, added into each reaction and incubated for 1.5 h at RT. Beads were removed with a magnet and then washed twice with 250 μl PBS with 0.2% SDS, twice with 250 μl low salt buffer (2 mM EDTA, 20 mM HEPES, 1% Igepal CA-630, and 150 mM NaCl), twice with 250 μl LiCl buffer (1 mM EDTA, 10 mM HEPES, 0.1% SDS, 0.1% SDC, and 250 mM LiCl), and once with 250 μl TE buffer (10 mM Tris and 1 mM EDTA). Bead complexes were transferred to a clean low binding 1.5 ml Eppendorf tube and then washed again with 250 μl TE buffer. Elution buffer (50 μl, 1% SDS) was added to each tube, and beads were boiled for 5 min followed by incubation at RT for 15 min. Supernatant was removed and added to a clean PCR tube. Then 170 μl elution buffer was added to each input and all samples, both input and eluates were decrosslinked for 16 h at 65°C. DNA was purified and analyzed as in the antibody-based ChIP protocol.

### Photomate enrichment of HDACs from crosslinked and sonicated cell lysates and western blotting

For enrichment experiments, 1 mg of crosslinked and sonicated cell lysate was diluted to 1 ml with PBS and treated with photomate, photomate ester or DMSO control for 20 min. Reactions were placed on a 12-well plate, cooled to 0°C and irradiated with 365 nm light (35 J/cm^2^). Samples were then incubated with azide conjugated biotin according to 1.5× concentration of probe, TCEP (0.25 mM), TBTA (50 μM), and CuSO_4_ (0.50 mM) for 90 min at RT. Samples were then left at **–**20 °C overnight. Precipitated protein was then spun down at 6000× *g* for 4 min at 4°C, and resuspended with brief sonication in 1 ml cold methanol. This was repeated twice and the pellets were resuspended in 1 ml 0.2% SDS in PBS by brief sonication and 10 min of heating at 60°C. Next, 80 μl Dynabeads M280 (Invitrogen) were washed twice with 0.2% SDS in PBS, added into each reaction and incubated for 1.5 h at room temperature. Beads were removed with a magnet and then washed twice with 0.2% SDS in PBS (500 μl), twice with 6 M urea (500 μl), twice with modified PBS with 500 mM NaCl and twice with PBS. Bound proteins were then suspended in 30 μl Laemmli buffer and boiled for 5 min. Beads were removed with magnet and samples separated by gel electrophoresis at 100 volts. Gels were transferred to nitrocellulose membranes with iBlot transfer system (P3 for 7 min), blocked with Odyssey blocking buffer (LiCor) for 2 h at 4°C, incubated with antibodies for class I HDACs overnight at 4°C, washed 3 × 5 min with PBST, incubated with anti-rabbit IRDye conjugated secondary antibody for 1 h and visualized with Odyssey Fc imager.

### Statistical analyses

Statistical analyses were performed with GraphPad Prism 6 software. All data are shown as mean ± SD. Student’s *t*-test (two-tailed) was used to measure statistically significant differences between groups. *P* value < 0.005 was considered statistically significant for this study.

## RESULTS AND DISCUSSION

In general, HDACi consist of (1) a zinc binding group (ZBG), (2) surface binding group (SBG), and (3) a linker to connect these two components and span the hydrophobic active site channel. The probe used in this study incorporates a photoreactive tetrafluorophenyl azide (TFPA) and an alkyne reporter into the SBG of the HDACi scaffold of suberanilohydroxamic acid (SAHA). The TFPA moiety can be used to covalently react with cellular targets of the photoreactive HDACi probe (photomate) and labeled proteins enriched after biorthogonal reaction of the alkyne reporter with an azide conjugated biotin tag. Although many photoreactive groups are synthetically accessible, the TFPA was chosen based on its similarity to aryl-based moieties present in the SBG of HDACi. In addition, the alkyne handle was used as it is small and stable in biological systems, yet reactive under copper(I)-catalyzed “click reaction” conditions. Recently, we characterized the activity and selectivity of photomate [16]. We found photomate has similar activity to the parent HDACi SAHA and could label HDAC1 and 2 but not other class I and II HDAC isoforms in MCF-7 cells. After demonstrating photomate’s ability to crosslink HDACs in cells, we envisioned it could be used to interrogate HDAC-chromatin interactions similar to an antibody-based ChIP experiment. A similar type of method has recently been developed to identify the genomic sites targeted by the bromodomain and extra-terminal motif (BET) inhibitor JQ1 [17]. However, this method depended on incorporation of a large biotin handle onto the parent BET inhibitor, which may affect both its activity and selectivity.

**Figure 1.**
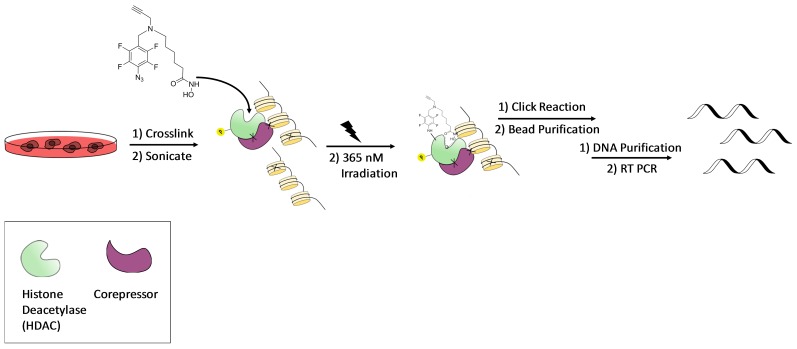
Graphical representation of photomate-based chromatin precipitation. Cells are double crosslinked with disuccinimidyl glutarate followed by formaldehyde and then sonicated. Protein-DNA adducts are incubated with photomate, UV-irradiated and reacted with an azide conjugated biotin tag *via* “click” chemistry. Chromatin is enriched with streptavidin coated beads followed by decrosslinking and DNA purification. Purified DNA is analyzed by PCR.

**Figure 2.**
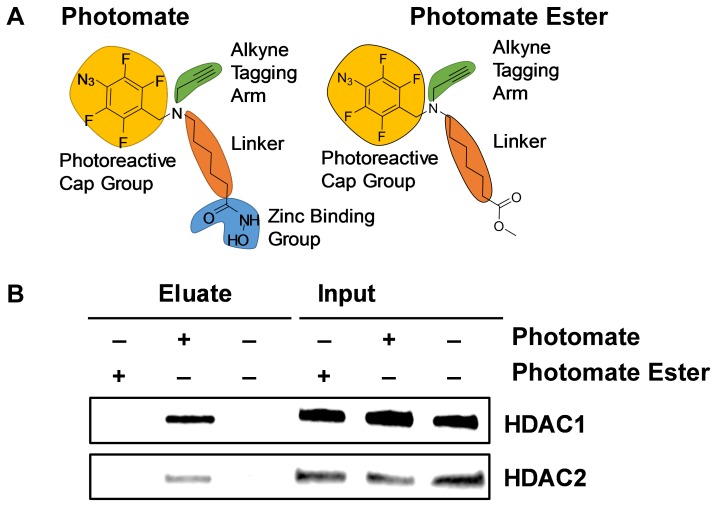
Photomate labels crosslinked and sonicated HDAC1 and 2. A. Structure of photomate and photomate ester control (photomate ester) lacking the zinc binding group. **B.** Western blot analysis of crosslinked and sonicated MCF-7 cells labeled with 10 μM photomate ester (lanes 1 and 4), 10 μM photomate (lanes 2 and 5), or DMSO only (lanes 3 and 6), reacted with biotin azide tag, and enriched with streptavidin coated magnetic beads. Enriched fractions were then decrosslinked and separated by electrophoresis. Blot shows enriched fraction eluted off of the beads and 2.5% of input recognized by anti-HDAC1 and 2 antibodies.

Typically, ChIP involves crosslinking chromatin bound proteins to DNA followed by sonication, lysis and immunoprecipitation with an antibody of interest. Enriched chromatin is then decrosslinked and the DNA is purified for quantification by PCR. **Figure 1** depicts the adaptation of ChIP experiment for use with photomate shown in **Figure 2A**. Similar to an antibody-based ChIP, chromatin is crosslinked and sonicated. As HDACs do not bind DNA directly, we found that a second crosslinking step in addition to the traditional formaldehyde crosslink was necessary to afford sufficient enrichment of canonical HDAC regulated genes. Necessity of the two-step crosslink was also observed previously [4,6,18]. Instead of antibody incubation, photomate is added to the crosslinked and sonicated cell lysates. After equilibration with photomate, bound HDAC targets are covalently attached *via* UV photolysis of the photoreactive TFPA moiety present in photomate’s scaffold. Labeled protein-DNA adducts are subsequently reacted *via* 1,3-Huisgen cyclization (“click reaction”) between the alkyne moiety on photomate and an azide conjugated biotin tag. We found that the cyclization reaction did not proceed efficiently in the presence of sodium dodecyl sulfate (SDS). This limits the use of SDS in the lysis buffer used for sonication. As SDS increases the efficiency of sonication, longer sonication times were necessary to achieve chromatin fragments of around 500 base pairs.

**Figure 3.**
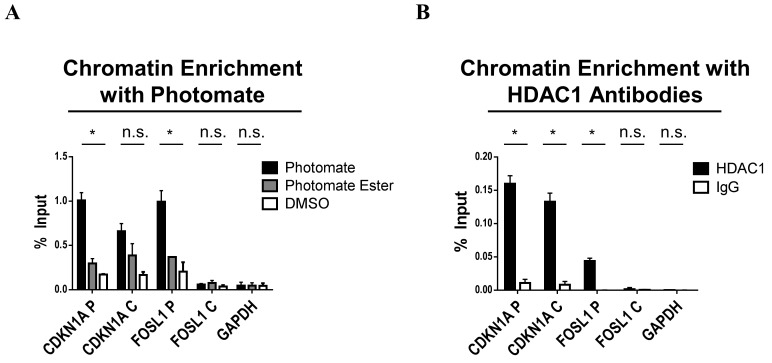
Photomate enriches HDACs associated with the CDKN1A promoter. **A.** Photomate significantly enriches CDKN1A and FOSL1 promoters, but not CDKN1A intron 1 FOSL1 Exon 1 or GAPDH in comparison to photomate Ester or DMSO Control. *P < 0.005. **B.** ChIP assay with HDAC1 antibody significantly enriches both CDKN1A and FOSL1 promoters and CDKN1A intron 1 but not FOSL1 Exon 1 or GAPDH promoter in comparison to IgG control. *P < 0.005.

After click reaction with biotin tag, photomate labeled adducts are enriched with streptavidin coated beads and eluted. As photomate is covalently bound to its targets, harsh washing conditions can be used to decrease non-specific protein interactions with the streptavidin coated beads. **Figure 2B** shows that the photomate enriches both HDAC1 and 2 with this procedure. Following elution, enriched protein-DNA adducts are decrosslinked, the DNA purified with a silica-based column, and the DNA sequences characterized by PCR. **Figure 3A** shows analysis of the CDKN1A and FOSL1 genes enriched by photomate in comparison to the control compound, photomate ester, and DMSO. We analyzed CDKN1A as it is one of the most well characterized HDAC1 and 2 occupied and regulated genes [19,20]. Additionally, HDAC occupancy of FOSL1 has been demonstrated several times [4,6]. Much like antibody-based ChIP utilizes an IgG antibody to control for non-specific enrichment of the antibody of interest, we utilized an ester of photomate (**Fig. 2A**). This control compound lacks the ZBG required to chelate the catalytic zinc ion in the active site of HDACs and thus should characterize the nonspecific binding and labeling with the TFPA. In addition, a DMSO control is added to inform on nonspecific click reaction products. For comparison, we also conducted a traditional ChIP experiment using a specific antibody for HDAC1 shown in **Figure 3B**. The main differences in these two experiments are that the HDAC antibody used in this study targets a specific portion of the c-terminus of HDAC1, while photomate targets the active site of multiple HDAC isoforms. Both antibody and photomate-based experiments show specific and significant enrichment of the promoter region of CDKN1A and FOSL1 in comparison to the promoter of GAPDH, which has been shown not to be regulated by HDACs [19,21,22]. We also analyzed a region spanning intron 1 of the CDKN1A gene as well as exon 1 of the FOSL1 gene. We observed that the HDAC1 antibody specifically enriched a significant amount of the CDKN1A intron 1 region, which was also reported by others [23]. However, photomate did not significantly enrich this region in comparison to the ester control. With respect to the FOSL1 gene body, neither photomate nor the HDAC1 antibody enriched this region.

The differences in antibody and photomate-based chromatin enrichment for the CDKN1A gene body suggests that those HDACs at the CDKN1A promoter are more catalytically competent than those in the gene body as the affinity of photomate for HDAC depends on the active site. This was experimentally examined in our recent publication where we found that binding of the probe to HDAC3 was increased when HDAC3 catalytic activity was increased by phosphorylation in comparison to control non-phosphorylated HDAC3 [16]. In addition, others have shown that phosphorylation of HDAC1 and 2 promotes enzymatic activity and that hyper-phosphorylated HDAC1 and 2 are more associated with gene promoters, while non-phosphorylated HDAC1 and 2 are associated with gene bodies [18,24]. Our method is able to detect these differences in HDAC activity at an individual gene of interest upon comparison to traditional HDAC antibody-based ChIP.

It is also interesting to note that the amount of chromatin enriched by photomate was on average 6.3 times higher in comparison to that enriched with the HDAC1 antibody, as reflected by the difference in percent of input pulled down by either method. These differences could result for several reasons. Others have shown multiple isoforms can be recruited to an individual promoter in MCF-7 cells [25]. As photomate labels multiple HDAC isoforms (**Fig. 2B**), it simultaneously enriches the chromatin associated with these isoforms, whereas the antibody would only capture the chromatin associated with one isoform; thus, more enrichment of the CDKN1A and FOSL1 promoters. Alternatively, the antibody could target a smaller subset of the total HDAC1 pool in comparison to photomate. This may result from limited access to the antibody epitope on the c-terminus, in comparison to the HDAC1 active site. Finally, since the CDKN1A intron 1 region shows some degree of non-specific enrichment with the ester control, ~0.4% input, this may indicate that photomate enriches more chromatin due to some amount of background, non-specific binding of photomate at the CDKN1A and FOSL1 regions evaluated. However, it should be noted that both the FOSL1 exon 1 and GAPDH regions, had very low non-specific enrichment, indicating the background labeling is loci specific. Regardless of the reason, the antibody-based method captures significantly less of the genes evaluated in this study in comparison to the photomate -based experiments.

In summary, we show that photomate can be used to determine the HDAC isoforms targeted by HDACi in a cell line of interest as well as the genomic loci associated with these isoforms. The covalent bond between photomate and HDACs enables stringent purification conditions with minimal loss of interactions between photomate and HDAC-DNA complexes. The experimental workflow is similar to traditional antibody-based ChIP and comparison of the two methods provides unique insight into whether catalytic competency is required at a given genomic locus. This method is anticipated to be useful on a genomic scale to determine the entire subset of genes targeted by an HDACi in a cell type of interest. Determination of the set of genes targeted by an HDACi will better clarify their mechanism of action and may help predict inhibitor efficacy.
